# The role of astrocytes in amyloid production and Alzheimer's disease

**DOI:** 10.1098/rsob.170228

**Published:** 2017-12-13

**Authors:** Georgia R. Frost, Yue-Ming Li

**Affiliations:** 1 Chemical Biology Program, Memorial Sloan Kettering Cancer Center, New York, NY 10065, USA; 2 Programs of Neurosciences, Weill Graduate School of Medical Sciences of Cornell University, New York, NY, USA; 3 Pharmacology, Weill Graduate School of Medical Sciences of Cornell University, New York, NY, USA

**Keywords:** Alzheimer's disease, astrogliosis, neuroinflammation, amyloid beta

## Abstract

Alzheimer's disease (AD) is marked by the presence of extracellular amyloid beta (Aβ) plaques, intracellular neurofibrillary tangles (NFTs) and gliosis, activated glial cells, in the brain. It is thought that Aβ plaques trigger NFT formation, neuronal cell death, neuroinflammation and gliosis and, ultimately, cognitive impairment. There are increased numbers of reactive astrocytes in AD, which surround amyloid plaques and secrete proinflammatory factors and can phagocytize and break down Aβ. It was thought that neuronal cells were the major source of Aβ. However, mounting evidence suggests that astrocytes may play an additional role in AD by secreting significant quantities of Aβ and contributing to overall amyloid burden in the brain. Astrocytes are the most numerous cell type in the brain, and therefore even minor quantities of amyloid secretion from individual astrocytes could prove to be substantial when taken across the whole brain. Reactive astrocytes have increased levels of the three necessary components for Aβ production: amyloid precursor protein, β-secretase (BACE1) and γ-secretase. The identification of environmental factors, such as neuroinflammation, that promote astrocytic Aβ production, could redefine how we think about developing therapeutics for AD.

## 1. Alzheimer's disease

Alzheimer's disease (AD), the most common form of dementia, is characterized by diminished cognitive function, specifically dysfunction of memory and judgement. With a rapidly ageing population, AD has become a major public health concern.

Pathologically, AD is marked by the presence of extracellular amyloid plaques, intracellular neurofibrillary tangles (NFTs) and gliosis [1] in the brain. The extracellular amyloid plaques are mainly composed of aggregated β-amyloid peptide (Aβ), whereas the NFTs are intracellular and are composed of hyperphosphorylated tau, a microtubule-binding protein [2]. Gliosis is a non-specific phenomenon that occurs in response to any injury to the CNS and involves the activation, and often proliferation, of glial cells. In AD, gliosis is marked by increases in activated microglia and reactive astrocytes near the sites of amyloid plaques [3]. Reactive astrocytes surrounding amyloid beta plaques contribute to the local inflammatory response and modulate calcium signalling [4,5].

## 2. Amyloid beta

The widely accepted amyloid cascade hypothesis states that AD is driven by Aβ accumulation [6]. It is thought that Aβ aggregates trigger a cascade of reactions, involving NFT formation, neuronal cell death, neuroinflammation and gliosis and, ultimately, cognitive impairment. It is important to note that Aβ exists in many forms: monomers, dimers, oligomers, fibrils and plaques [7].

The amyloid cascade hypothesis has significant genetic support. Autosomal dominant AD (ADAD) mutations have been identified in amyloid precursor protein (APP) [8] and presenilin (PS) [9,10], two necessary genes for Aβ production. Specifically, 30 APP mutations, 9 APP duplications, 211 PS1 mutations and 33 PS2 mutations have been identified that cause early onset ADAD [11]. Furthermore, Down's syndrome patients with trisomy of chromosome 21 and individuals with small internal chromosome 21 duplications have an additional copy of APP and greatly increased risk of developing AD [12]. While these mutations provide insight into disease aetiology, they account for a very small percentage of AD cases. In addition, an APP mutation that reduces Aβ production protects against AD and age-related cognitive decline [13], providing another line of support for the Aβ hypothesis. It is worth mentioning that how these PS1 and PS2 mutations contribute to the pathogenesis of AD, such as if they are loss-of-function or gain-of-function, is the subject of considerable debate [14–19].

**Figure 1.**
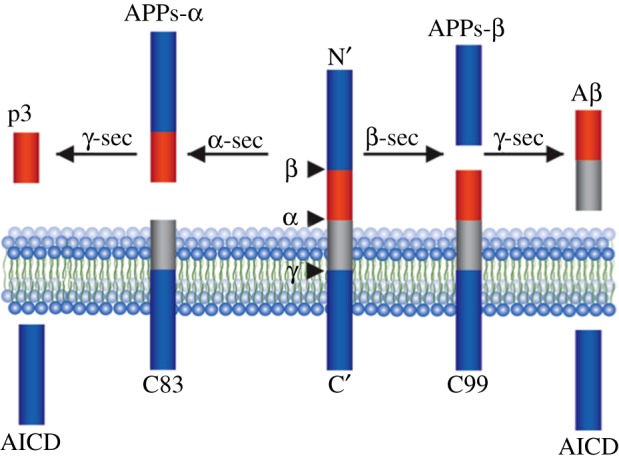
Aβ production. In the amyloidogenic pathway (right), APP is cleaved by BACE followed by γ-secretase which releases Aβ peptides and APP intracellular domain (AICD), which generate the N and C termini of Aβ, respectively. In the non-amyloidogenic pathway (left), APP is cleaved sequentially by α-secretase and γ-secretase, which does not result in the generation of Aβ species.

Most commonly, AD presents as a sporadic multi-factorial condition. ADAD studies strongly indicate that Aβ plays a critical role in AD pathogenesis. APP is a highly conserved integral membrane protein thought to play a role in synapse formation and neural plasticity, but its primary function has yet to be described. It can be processed in two separate pathways. In the amyloidogenic pathway, APP is first cleaved by β-secretase (BACE) followed by γ-secretase cleavage and release of Aβ peptides and APP intracellular domain (AICD) (figure 1) [20–23]. In the non-amyloidogenic pathway, α-secretase and then γ-secretase cleave APP sequentially; this does not result in the generation of Aβ species and acts as a negative feedback on γ-secretase activity [24,25].

BACE1, an aspartyl protease, is tethered to the membrane by a long tail; it is found in the endoplasmic reticulum and Golgi and functions to prune proteins. Many of its substrates are involved in neural function, including neuregulin and voltage-gated sodium channels [20–23,26].

PS is the catalytic subunit of γ-secretase [27,28]. Three other necessary γ-secretase subunits have also been identified: nicastrin (Nct), anterior pharynx-defective-1 (Aph1) and presenilin enhancer-2 (Pen2) [29,30]. These four components constitute the mature γ-secretase complex [31,32], and their stepwise assembly, followed by endoproteolysis of PS into amino-terminal (PS-NTF) and carboxy-terminal fragments (PS-CTF), is necessary for active complex formation [33]. Therefore, γ-secretase activity is regulated by the abundance of the four essential subunits and their assembly. Additionally, only a small fraction of γ-secretase in the cell is actually catalytically active [34–37]. This suggests that additional events are necessary to activate the inactive complex [38].

## 3. Astrogliosis and neuroinflammation

Aβ accumulation triggers a neuroinflammatory state that plays a significant role in the progression of AD [39,40]. Levels of Aβ in the brain are regulated by an innate immune response. Aβ, thought to be primarily produced by neurons, can activate an inflammatory response that ultimately drives microglia and astrocytes to uptake and clear it from the brain [41–43]. Genetic studies identifying single-nucleotide polymorphisms in inflammatory genes that are associated with the risk of AD underline the involvement of inflammation in AD [44–48]. Furthermore, AD patients have higher levels of proinflammatory cytokines and activated inflammasomes [49].

Astrocytes are key regulators of the brain's inflammatory response and, as mentioned previously, reactive astrogliosis is a universally acknowledged feature of AD. Marked by cellular hypertrophy and an increase in glial fibrillary acidic protein (GFAP) and S100B expression, astrogliosis is observed in post-mortem tissues from AD patients and mouse models. Moreover, the degree of astrogliosis is correlated with cognitive decline [50–52].

Astroglia are found throughout the CNS and are thought to be the most prevalent cell type in the brain [53]. Astrocytes function in territorial domains in which they are connected to the vasculature through processes terminating in the endfoot. Furthermore, their processes also envelope neuronal synapses [54]. This intricate system of connections enables astrocytes to exert control over many necessary brain functions including regulation of the blood–brain barrier, delivering nutrients to nervous tissue and maintaining ion and metabolite balance. Astrocytes can propagate calcium currents, release gliotransmitters and signal with neurons [55]. Specifically, astrocytes release neurotransmitters such as glutamate, GABA and ATP, neuromodulators d-serine and kynurenic acid, and growth factors and inflammatory mediators [56–58]. A chief role of astrocytes in the brain is to protect in all manners against CNS injury and to repair nervous tissue after injury. This is primarily achieved through astrogliosis, an evolutionarily conserved event that contributes to the neuroprotection and isolation of damaged tissue through the formation of a glial scar and removal of pathogens from the CNS [59].

**Figure 2.**
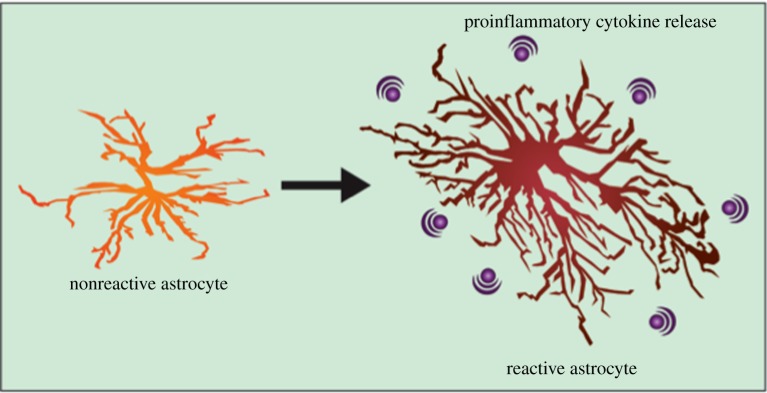
Cellular stress can trigger astrogliosis—increased numbers of reactive astrocytes, which are characterized by hypertrophy of processes. Astrocytes undergo many molecular changes when activated and can secrete a plethora of proinflammatory cytokines.

Astrogliosis occurs when astrocytes respond to injuries to the CNS by undergoing a spectrum of molecular and morphological changes. Inflammatory mediators released by microglia, neurons, oligodendrocytes, endothelial cells, leucocytes and other astrocytes in response to injury initiate the changes associated with an astrocyte becoming reactive. Molecular changes entail a wide spectrum of genes resulting in differing expression of structural proteins, transcriptional regulators, extracellular matrix components, inflammatory regulators, vascular regulators and synaptic modulators (figure 2).

Interestingly, these molecular changes are highly context specific. While there is a core group of genes that are consistently upregulated across different reactive models, approximately 50% of the altered gene expression varies depending on the initiating injury [60,61]. The primary morphological change during reactive astrogliosis is hypertrophy of processes, which is linked to increased expression of intermediate filament, most notably GFAP [62,63]. The functional consequences of this increased GFAP expression are not yet fully understood; however, it appears to be critical in limiting Aβ plaque build-up.

The impact of reactive astrogliosis in disease is complex: reactive astrocytes can be both harmful and beneficial to surrounding cells and may worsen or resolve the initial CNS injury. Notably, reactive astrocytes are necessary for scar formation, which helps to contain the spread of inflammatory cells, and also for repairing insults to the blood–brain barrier. Reactive astrocytes surround Aβ plaques in a manner similar to glial scarring and express receptors such as RAGE, low-density lipoprotein receptor-like protein, membrane-associated proteoglycans and scavenger receptor-like receptors that are known to bind Aβ [3].

Conversely, reactive astrocytes may be neurotoxic when producing reactive oxygen species or some inflammatory cytokines [64]. Further research into the mechanisms regulating the balance between when reactive gliosis is neuroprotective and when it is neurotoxic is critical to understanding the functional consequence of reactive astrocytes in AD. However, a caveat to much of this research is the reliance on rodent models, as human astrocytes are much larger and have a multitude more processes than their rodent counterparts [65]. To summarize, reactive astrogliosis is a complicated and diverse phenomenon yet is ubiquitous across various CNS pathologies.

## 4. Astrocytes in Alzheimer's disease

Astrocytes undergo complex and conflicting region-specific changes during the course of AD. The number of astrocytes is thought to remain constant throughout the brain during the progression of disease; however, some populations, those in proximity to amyloid beta plaques, become reactive, while conversely, large numbers of astrocytes atrophy [66]. In the 3XTg AD model, the initial astroglial phenotype is general dystrophy [66].

Astrodegeneration, defined as reduced astrocyte volume and surface area and a decrease in protoplasmic processes, has been observed in mouse models of AD. Beginning early in pathology, prior to amyloid beta presence, 3XTg AD mice exhibit astrodegeneration in the medial prefrontal cortex (mPFC), entorhinal cortex (EC) and hippocampus [3,67,68]. Similarly, astroglial atrophy is observed in the hippocampus prior to amyloid beta presence in the PDAPP mouse model [69]. At the later stages of disease, the presence of Aβ triggers a secondary astroglial response by activating astrocytes, resulting in reactive astrogliosis in areas surrounding the plaques [3,70].

Both resting and reactive astrocytes are key regulators of the brain's inflammatory response and are capable of releasing, and responding to, a spectrum of immune mediators [71]. Specifically, astrocytes secrete many cytokines capable of inducing inflammation, notably IFN*γ*, IL-1β, TNF*α*, IL-6 and TGFβ [72–75]. Many of these proinflammatory cytokines are upregulated in human AD brain samples and in transgenic mouse models of AD [76–79].

IFN*γ* is a potent regulatory cytokine that activates microglia, promotes inflammation and is upregulated in the AD brain [80]. It is primarily produced by T cells and natural killer cells but can also be secreted by microglia and astrocytes [81,82]. TNF*α*, a cytokine involved in inducing acute-phase inflammation, is elevated in AD serum, CSF and cortex [83]. Tg2576 mice deficient in CD40 (a TNF receptor gene) have reduced BACE activity, Aβ load and gliosis compared with normal Tg2576 mice, highlighting the importance of TNF in AD progression [79]. IL-6 can be both proinflammatory and anti-inflammatory and has been reported to be elevated in the plasma, cerebrospinal fluid, and the brain of AD [84–89]. IL-1β, one of the first cytokines secreted in response to injury, is an important mediator of inflammatory response as well as cell proliferation, differentiation and apoptosis. It is found at high levels near the sites of amyloid plaques [87,90,91].

A genetic polymorphism in transforming growth factor β1 (TGFB1), an immunosuppressive cytokine, is associated with the risk of developing AD [92]. Additionally, post-mortem AD brains contain increased levels of TGFβ, specifically in plaques, suggesting it may play a role in pathology [93,94]. In agreement, aged mice overexpressing TGFβ in astrocytes displayed Aβ deposition, and astrocytes containing TGFB1 are found in close proximity to Aβ deposits in mice overexpressing APP with the Swedish mutation. This suggests that the mechanism by which TGFβ contributes to pathology is astrocyte specific [78,95–97].

## 5. Astrocytes contribute to Aβ load

It was long thought that neurons were the only cell type that expressed high levels of BACE1 and, therefore, were the only cell capable of producing Aβ [98]. However, subsequent studies have demonstrated that astrocytes express BACE1 at sufficient levels to generate Aβ, and that expression can be increased by cellular stress [99–104]. Additionally, stressors can upregulate APP expression and, therefore, Aβ secretion. The effect of cellular stress on the activity of γ-secretase, the third necessary factor for amyloid production, in astrocytes has yet to be fully elucidated. As astrocytes substantially outnumber neurons in the brain, the identification of environmental factors (i.e. inflammation), which promote astrocytic Aβ production, could redefine how we think about developing therapeutics for AD.

As mentioned previously, FAD mutations in APP and PS have been extensively studied to gain insight into the mechanisms underlying AD. However, the majority of these studies have focused on neurons. In order to determine the effect of FAD PS1 mutations on Aβ production from cell types other than neurons*,* Veeraraghavalu *et al*. [105] selectively inactivated PS1*Δ*E9 in postnatal forebrain excitatory neurons, which are thought to be the primary source of Aβ, by crossing PS1*Δ*E9flox mice with CaMKIICre mice. They determined that at 10–12 months of age, total Aβ burden in these mice was indistinguishable from mice expressing PS1ΔE9 in all cell types. This suggests that the FAD mutation drives high Aβ load by increasing Aβ secretion in other types of neurons or glial cells. To identify these other cellular sources of Aβ, they dissociated primary astrocytes and microglia from the brains of newborn PS1*Δ*E9flox or APP^swe^ mice or 8-week-old APP^swe^/PS1*Δ*E9flox mice. Both astrocytes and microglia secreted detectable Aβ. Treatment with a γ-secretase inhibitor prevented Aβ secretion and resulted in the accumulation of the βCTF fragment [105]. This suggests that not only are astrocytes capable of producing Aβ, but that they do so in levels substantial enough to contribute significantly to total amyloid load. If this is the case, then astrocytes must express appreciable levels of APP, BACE and γ-secretase. In support, Grolla *et al*. [106] detected APP, BACE1 and γ-secretase subunits PS1, PS2, PEN2 and NCT expression in primary rat hippocampal astrocyte cultures.

## 6. Amyloid precursor protein expression in astrocytes

APP is expressed in all tissues; however, the relative amount of APP in different cell types varies [107–109]. Astrocytic expression of APP has been demonstrated. APP^695^, APP^751^ and APP^770^ mRNA have been identified in non-neuronal cells in the human brain [110] and rat astrocytes [111]. This is supported in primary microglial and astrocyte culture of newborn rat pups, which express mRNA for all three APP isoforms [112]. When normalized to beta-actin mRNA levels, primary rat astrocytes expressed 94% of the amount of APP as neurons [113]. Additionally, inflammatory mediators have been shown to regulate APP levels [114–116].

Multiple proinflammatory cytokines have been shown to upregulate APP in the mouse brain and in human neuroblastoma cells and non-neuronal cells such as human astrocyte cultures [116]. This implies that in the neuroinflammatory context of AD, reactive astrocytes express higher levels of APP than when at rest and, therefore, could produce more Aβ. Lipopolysaccharides (LPS) treatment induces chronic neuroinflammation [117,118] and can contribute to learning and memory deficits [119–121]. It has been established that stress from injections of LPS, a known activator of CNS glia [122], can induce a twofold increase in APP expression in the whole brains of APP^swe^ mice. Dramatically, LPS treatment resulted in an 18-fold increase in βCTF, suggesting massively increased BACE activity, and ultimately a threefold upregulation of both Aβ40 and Aβ42. While this study looked at the whole brain, and not specific cell types, it is worth noting that LPS treatment also increased the levels of GFAP-positive astrocytes in the cortex and hippocampus [123].

APP expression can be upregulated by the transcription factor AP-1, which is found in the promoter region of most acute-phase proteins that are induced by IL-1β and IL-6, suggesting APP may be regulated by these specific cytokines [114,115]. Supporting this, IL-1β has been shown to upregulate APP in human astrocytes and the U373MG human astrocytoma cell line [124,125].

Zhao *et al.* [103] demonstrated that in primary mouse astrocytes, stimulation with several proinflammatory cytokine combinations (LPS + IFN*γ*, TNF*α* + IFN*γ* and TNF*α* + IL-1β + IFN*γ*) markedly increases expression of APP. The same combinations of cytokines also induce increases in BACE1 protein by up to eightfold. The downstream consequence is a 20–40% increase in Aβ40 secretion [103]. A combination of IFN*γ* and TNF*α* has also been shown to induce Aβ secretion from primary human astrocytes and the U373 cell line [104]. To conclude, systemic inflammation from LPS treatment and specific AD-associated inflammatory mediators can upregulate APP expression in astrocytes.

As previously mentioned, TGFβ is associated with AD development [92]. Lesné *et al.* [126] confirmed this effect in a mouse model that overexpresses TGFβ under the GFAP promoter, driving astrocytic expression. They found increased APP and soluble Aβ40 and Aβ42 in the whole brain. To investigate whether the increase in Aβ was produced by the astrocytes, or whether the TGFβ overexpressing astrocytes released a secondary mediator that, in turn, induced neuronal Aβ production, they cultured primary neurons and astrocytes with TGFβ. They found an increase in APP and Aβ40 and Aβ42 in the astrocyte culture, but not in the neuronal culture, indicating that the increased Aβ in the mice was produced by astrocytes [126]. Furthermore, astrocytoma cell lines and normal human astrocytes have increased expression of APP when exposed to TGFβ [127–129]. Taken together, this suggests that TGFβ increases Aβ in the AD brain by inducing APP upregulation in astrocytes and subsequently inducing astrocytic Aβ secretion.

## 7. BACE1 expression in astrocytes

It has been thought that astrocytes do not contribute to Aβ load due to a lack of BACE1 activity, which is highly expressed in the brain but primarily in neurons [22]. There is little evidence to suggest that nonreactive astrocytes express significant levels of BACE1 [99,130,131]. Zhao *et al*. [132] generated two mouse models overexpressing the APP^751^. In one line, APP was under the NSE promoter driving neuronal expression, while in the other line the GFAP promoter was used to drive astrocytic expression. They determined that primary neurons from the NSE-APP mice produced large amounts of βCTF, suggesting high BACE1 activity. However, primary astrocytes from the GFAP–APP mice had very little BACE1 activity and no detectable Aβ production [20–23,132].

Studies propose that resting astrocytes express BACE1 mRNA but not protein, indicting a translational block may inhibit Aβ production in astrocytes [131]. This suggests that stress may be able to upregulate BACE1 activity by overturning this translational block. Hartlage-Rubsamen *et al.* [130] demonstrated that activation of glial cells induces BACE1 expression in six rat models of acute stress known to induce reactive gliosis: LPS + IFN*γ* treatment, intraaccumbal α,β-methylene adenosine 5′-triphosphate (α,β-meATP) treatment, middle cerebral artery occlusion, experimental autoimmune encephalomyelitis or Borna disease virus infection. Interestingly, they found marked increases in BACE1 expression in GFAP-positive astrocytes in the chronic models of stress, but not in the acute inflammation resulting from LPS + IFN*γ* or α,β-meATP [130]. This suggests that in the context of AD, neuroinflammatory stress my upregulate BACE expression in astrocytes. In fact, BACE1 expression has been demonstrated in reactive astrocytes and in astrocytes in human AD patients [99–102].

BACE expression is observed in reactive astrocytes around amyloid plaques. The number of these BACE1-positive reactive astrocytes was increased in AD patients compared to old age controls, particularly in the entorhinal cortex [100]. In Tg2576 mice, which overexpress APP^695^ with the Swedish mutation, the level of BACE1 protein is correlated to the level of Aβ in astrocytes. Additionally, reactive astrocytes surrounding Aβ plaques always stain positively for BACE1 protein, whereas resting astrocytes do not [99,101].

Similar to APP expression, inflammation has also been shown to upregulate BACE1 [133]. Specifically, proinflammatory cytokines upregulate BACE1 activity [134]. Many proinflammatory cytokines signal through JAK/STAT pathways to ultimately influence transcriptional changes. STAT1 directly binds to the BACE1 promoter, suggesting a possible mechanism by which inflammation induces BACE1 expression. Specifically, IFN*γ* induces BACE1 expression in the U373 cell line and in primary mouse astrocytes [135]. In support of this, it has been shown that IFN*γ* and TNF*α* regulated BACE1 expression and Aβ production in APP^swe^ transgenic mice. Furthermore, APP overexpressing mice, with the IFN*γ* receptor knocked out, have reduced Aβ deposition compared with APP transgenic mice. This is paired with reduced numbers of astrocytes and microglia in the cortex and hippocampus. Primary astrocytes overexpressing APP with the Swedish mutation (via adenovirus) secrete higher levels of TNF*α* than wild-type (WT) astrocytes, and this effect is abolished in IFNGR KO astrocytes, indicating that IFN*γ* signalling is critical for TNF*α* secretion. Furthermore, TNF*α* induces BACE1 expression and Aβ production in astrocytes in a dose-dependent manner. This effect was enhanced by the addition of IFN*γ* [136]. Taken together, we can conclude that IFN*γ* and TNF*α* upregulate BACE1 expression in astrocytes and ultimately increase Aβ secretion. Subsequently, Cho *et al.* [137] demonstrated that this upregulation was mediated by the activation of JAK2 and ERK1/2 signalling.

Other inflammatory mediators have also been shown to upregulate BACE1 expression. NF-κB is a protein complex of DNA transcription factors that plays a role in cytokine production and cell survival. In the aged and AD brain, there are increased levels of NF-κB and NF-κB transcription factor-mediated responses to stress are enhanced [138–140]. Aβ stimulates NF-κB activation in primary rat astrocytes in a dose- and time-dependent manner [141]. The rat and human BACE1 promoters have an NF-κB binding site [142]. Deletion studies suggest that the NF-κB binding site suppresses BACE1 expression and Aβ secretion in neurons when occupied by NF-κB [143,144]. It has been demonstrated that NF-κB suppresses BACE1 expression in nonreactive astrocytes; however, it has the opposite effect in TNF*α*-activated primary rat astrocytes. We can conclude, that in the context of inflammation, when astrocytes are reactive, NF-κB can induce BACE1 expression [145].

Other specific stress-induced pathways that upregulate BACE1 have been identified. The transcription factor Ying Yang 1 (YY1) functions in glucose metabolism, DNA repair and notch signalling and can bind to the BACE1 promoter to induce BACE1 activity in primary rat astrocytes and neurons. When permanent cerebral ischaemia was induced in rats by coagulation of the middle cerebral artery [146] in order to stress astrocytes, primary cultured astrocytes from these rats robustly expressed YY1. This suggests that YY1 can upregulate BACE1 activity in astrocytes under stressful conditions [147].

Furthermore, astrocytes also express BACE2, a close homologue of BACE1. Whether BACE2 activity results in APP cleavage, and ultimately Aβ production, is still up for debate. Ablation of BACE1 and BACE2 in a mouse model had reduced Aβ production compared to just a BACE1 knockout, suggesting BACE2 does, in fact, contribute to Aβ load [148]. BACE2 activity is detectable in nonreactive primary rat astrocytes and levels of activity actually decrease when the astrocyte is activated [103,104,131].

Aβ itself could be considered a proinflammatory mediator due to its ability to induce inflammation [149]. Additionally, it is well established that Aβ can stimulate proinflammatory cytokine release from astrocytes [150,151] Therefore, we can conclude that in AD, Aβ itself can upregulate BACE1 expression in astrocytes by stimulating an inflammatory response. Additionally, Aβ may cause neurotoxicity by disrupting intracellular calcium homeostasis in neurons and in glial cells. Disrupted calcium homeostasis is observed in the brains of AD patients; however, the mechanism of this Aβ-induced deregulation is unclear [152]. Nuclear factor of activated T cells (NFAT) is a transcription factor that regulates BACE1 expression by directly binding to its promoter region in response to signalling by the calcium- and calmodulin-dependent phosphatase calcineurin. BACE1 expression is enhanced in primary neuronal cells and SH-SY5Y neuroblastoma cells after stimulation by a calcium ionophore. This upregulation can be blocked by pretreatment with either an inhibitor of calcineurin or a calcium chelator. Aβ treatment stimulates activation and nuclear translocation of NFAT1 resulting in increased BACE1 expression. Additionally, NFAT1 activation is observed in APP^swe^ mouse brains. Taken together, Aβ induces increases in intracellular calcium that can stimulate BACE1 expression, inducing further Aβ generation [153].

Jin *et al*. [101] demonstrated Aβ1–42 or Aβ25–35 treatment enhances BACE1 promoter activity and BACE1 protein levels in U373 cells, and this can be blocked by pretreatment with a calcineurin inhibitor. This increase in BACE1 levels resulted in increased Aβ secretion that could also be prevented by pretreatment with a calcineurin inhibitor. Furthermore, this Aβ-induced BACE1 upregulation can be blocked by preventing calcium influx through treatment with 2APB, an inhibitor of IP_3_-dependent calcium release, and U73122, an inhibitor of PLC. Aβ can form pores in cell membranes that may be permeable to calcium influx, which can be blocked by Zn^2+^ [154]*.* Jin *et al.* [101] used pretreatment with ZnCl_2_ to prevent calcium influx through Aβ-induced pores and found that Aβ no longer enhanced BACE1 expression.

Dal Prà *et al*. [155] found little Aβ secretion from resting normal adult human astrocytes. However, when these cells are activated by exposure to Aβ25–35, an Aβ42 proxy which contains its active site [155], there was a translocation of HIF*α* to the nucleus. This upregulated BACE1 and increased γ-secretase activity, ultimately leading to significant Aβ42 secretion [156]. These observations indicate that neuronally secreted Aβ could induce Aβ production in reactive astrocytes through HIF1*α*.

## 8. γ-Secretase activity in astrocytes

As previously mentioned, γ-secretase activity is not correlated with the quantity of catalytic subunit; this makes it difficult to quantify, so it is not surprising that little is known about γ-secretase activity in astrocytes in AD. PS1 mRNA is highly expressed in astrocytes [10], and PS1 protein expression has been confirmed in glial cells in primate brain; however, staining is weak compared to neuronal cells [157]. Similar to APP and BACE1 expression, PS1 is elevated in reactive astrocytes in the AD brain [158,159]*.* Specifically, TGFβ may upregulate PS1 mRNA in the human U87 MG astrocytoma cell line [160]. However, as γ-secretase protein levels do not correlate to γ-secretase activity levels, these studies do not fully elucidate the role of γ-secretase-mediated Aβ production in astrocytes.

## 9. Other stressors

In addition to neuroinflammation, there may be other physiologically relevant cellular stressors that trigger APP and BACE and promote Aβ generation in astrocytes. Under stress, the activation of the hypothalamic–pituitary–adrenal axis results in glucocorticoid (GC) secretion from the adrenal cortex. Elevated GC is associated with cognitive impairment and has been implicated in AD pathology [161,162]. There are GC response elements in the APP and BACE1 promoter, indicating that GC signalling can upregulate their expression [163,164]. Wang *et al*. [165] demonstrated that GCs promote Aβ40 and Aβ42 secretion from primary mouse astrocytes. They attributed this to an increase in both APP and BACE1 mRNA and protein. Next, they demonstrated a similar increase in APP, BACE and Aβ production in 9-month-old mice treated with dexamethasone. Furthermore, this treatment induced reactive astrocytes that stained positively for both APP and BACE, indicating that this change occurs primarily in reactive cells [165].

Other stressors, such as tissue damage, have been shown to induce APP expression in astrocytes. Hippocampal lesions stimulate APP expression in nearby astrocytes [166]. Brain injury has been shown to enhance astrocytic APP expression [167,168]. Traumatic brain injury has long been linked to the risk of developing AD [169,170] and is associated with accelerated Aβ deposition in AD [171]. Significant evidence suggests that acute brain injury can induce PS1 expression in mice [172], rats [173] and in human brains following cerebral infarcts [174,175]. Importantly, these studies indicate that brain injury induces PS1 expression in astrocytes. Nadler *et al.* [176] used three models to induce brain injury closed head injury (CHI), a well-established model for head trauma which is accompanied by neuroinflammation [177], brain stabbing or intracerebroventricular injection of LPS. In each incidence, the brain trauma resulted in more reactive astrocytes and increased expression of presenilin-1 and nicastrin.

Inorganic arsenic (iAs), a toxic metalloid, can contaminate drinking water and is associated with cognitive impairment [178,179]. Cells process iAs to a highly toxic monomethylarsonous acid, MMA^III^, which has been suggested to be associated with neurodegenerative disorders, although epidemiological studies have failed to demonstrate association. Primary rat astrocytes exposed to MMA^III^ have increased mRNA levels of a plethora of AD-related cytokines including IL-1β, IL-6 and TNF*α*. Additionally, MMA^III^ induces a roughly threefold increase in APP and BACE1 mRNA expression [180].

**Figure 3.**
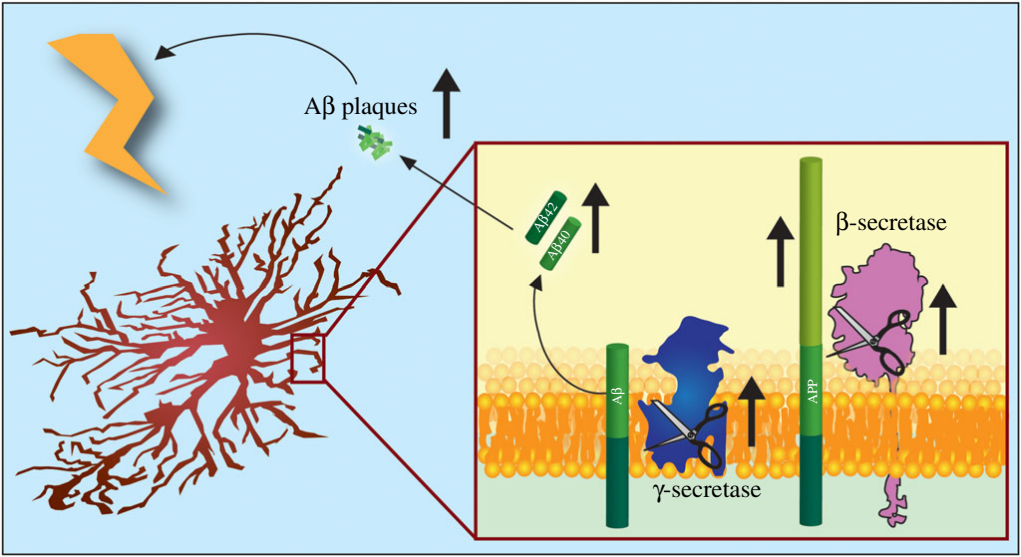
A feed-forward mechanism of Aβ secretion by reactive astrocytes. Cellular stressors and proinflammatory cytokines upregulate APP, BACE and γ-secretase in astrocytes resulting in astrocytic Aβ production. In turn, this Aβ initiates further stress and inflammation driving subsequent Aβ production.

These studies fit with the wider narrative that chronic stress can induce Aβ production [181,182]. Taken together, this suggests that a feed-forward mechanism is at play. Amyloid beta, perhaps initially from neurons, stimulates proinflammatory cytokine release from microglia and astrocytes, which in turn leads to upregulation of APP and BACE expression and possibly γ-secretase activity to drive astrocytic Aβ secretion (figure 3). This inflammation-induced Aβ then stimulates further neuroinflammation and ultimately additional amyloid production.

Furthermore, Aβ produced by astrocytes may be more pathogenic than that of neurons. A large portion of the Aβ species comprising amyloid beta plaques are N-truncated [183–185]. Aβ peptides beginning at Glu3 and at Phe4 are abundant in plaques. Studies suggest that these N-truncated species arise because other enzymes either compete with or modify BACE1 cleavage of APP [186–188]. The proportion of N-truncated peptides making up Aβ plaques seems to increase with disease progression and Braak stage [189]. N-truncation may affect the pathogenicity of the peptide; for example, Aβ42 with a truncated N-terminus is highly prone to aggregation [190]. Oberstein *et al.* [191] observed that astrocytes produced sevenfold less Aβ40 than neurons; however, they found that 60% of the Aβ secreted by astrocytes was N-truncated, compared to 20% from neurons.

## 10. Astrocytes and ageing

As the primary risk factor for AD is ageing, it is important to understand the effect of ageing on astrocytes. However, currently, little is known about the effect of ageing on metabolic, biochemical and morphological changes in astrocytes. Some research has demonstrated that ageing is associated with increased astroglial proliferation and reactivity, measured as an increase in GFAP expression, particularly in the CA1 region of the hippocampus [192–196]. In this model of ‘inflammageing’, reactive astrocytes contribute to chronic neuroinflammation throughout the brain [197]. This suggests that there is the potential for ageing to induce reactive astrogliosis, which could result in astrocyte Aβ production. Therefore, ageing-induced neuroinflammation may be an initiating factor of late onset AD.

Studies in other regions of the brain have found evidence of age-associated astroglial atrophy [198–200]. Using GFAP, glutamine synthetase, and s100β as markers for astroglia, Rodriguez *et al*. [66] confirmed ageing is associated with both reactivity and atrophy in different brain regions. Owing to the heterogeneity of astrocytes throughout the brain, it is possible for ageing to be associated with both astroglial dystrophy and reactivity. To conclude, ageing-dependent changes in astroglia are context and region specific, and more research into the downstream consequences of astroglial ageing needs to be done as a framework to fully understand the role of astrocytes in AD.

## 11. Aβ as an anti-microbial protein

Aβ is generally described as having no normal physiological role. However, it has recently been suggested that Aβ may act as anti-microbial protein (AMP) in the brain as first line of defence against invading pathogens. AMPs, also known as host defence peptides, are broad spectrum antibiotics that are active against a host of pathogens, including bacteria, fungi and viruses [201]. If this is the case, then astrocytes, as mediators of innate immunity, may secrete Aβ in response to stress as an innate defence mechanism.

BACE1 and BACE2 double knockout mice have higher neonatal mortality rates than WT mice and this is not due to maternal care issues or deficient active immunity. This increased mortality disappears when the mice are housed in a pathogen-free facility, suggesting they may have increased susceptibility to pathogens [148]. If this is the case, their compromised innate immune system may be due to the lack of Aβ in these mice.

Soscia *et al.* [202] compared the ability of Aβ to inhibit the growth of pathogens to that of LL-37, an established human AMP in the cathelicidin family [202,203]. Aβ was active against 8 of 12 pathogens, in rates similar to that of LL-37. Furthermore, homogenate from the temporal lobe of AD patients had 24% greater activity against *Candida albicans* than that of control subjects. This activity returned to that of control patients when they immunodepleted Aβ from the homogenate [202].

Aβ has also been shown to be protective against viral infections such as Herpes simplex virus 1 (HSV-1) and H3N2 and H1N1 influenza A virus (IAV). HSV-1 is a known AD risk factor and viral particles colocalize with amyloid plaques [204,205]. Pretreatment of fibroblast, epithelial and neuronal cell lines with either Aβ40 or Aβ42 reduced HSV-1 replication [206]. *In vitro,* Aβ42 reduces epithelial cell uptake of IAV and causes aggregation of viral particles. Aβ42 also reduced viral protein synthesis monocytes and decreased IL-6 secretion [207].

After intracerebral injection of *Salmonella* Typhimurium, 4-week-old 5XFAD transgenic mice, which overexpress both human APP and PS1 with five FAD mutations, and therefore express high levels of Aβ, had increased survival compared with WT mice. In the same assay, APP knockout mice that do not produce Aβ fared worse than non-transgenic mice. Consistent with this, the human brain neuroglioma cell line H4, stably overexpressing either Aβ40 or Aβ42, has increased survival when challenged with *C. albicans* compared to WT H4 cells. The mechanism behind this seems to be reduced adhesion to Aβ overproducing cells and increased microbial agglutination [208]. Taken together, this suggests that Aβ may play a role as an AMP in the innate immune system and this may explain why astrocytes secrete Aβ in response to cellular stressors.

## 12. Interplay with neurons and microglia

If inflammation-induced astrocytic Aβ production plays a significant role in AD pathology, then the relationship between microglia, astrocytes and neurons needs to be redefined. Microglia play a central role in the immune system of the CNS and produce and respond to a variety of inflammatory mediators that are implicated in AD [209]. Genetic studies support the significance of microglia in pathology: it is well established that mutations in triggering receptor expressed on myeloid cells 2 (TREM2) and CD33 increase the risk for AD [45–48,210].

Similar to astrocytes, microglia undergo substantial changes in response to stimulus and may be resting or activated depending on the cellular environment [211]. Microglia can be activated by Aβ from either neurons or astrocytes and in response secrete proinflammatory cytokines [212,213]. It has been suggested that there is a delicate balance between harmful and beneficial microglial cytokine production in AD. Inflammation is necessary to promote efficient microglial clearance of Aβ, but excessive inflammation may accelerate disease by causing neuronal and glial cell death [214,215]. We argue that microglial cytokine production also contributes to disease progression by inducing astrocytic Aβ production.

**Figure 4.**
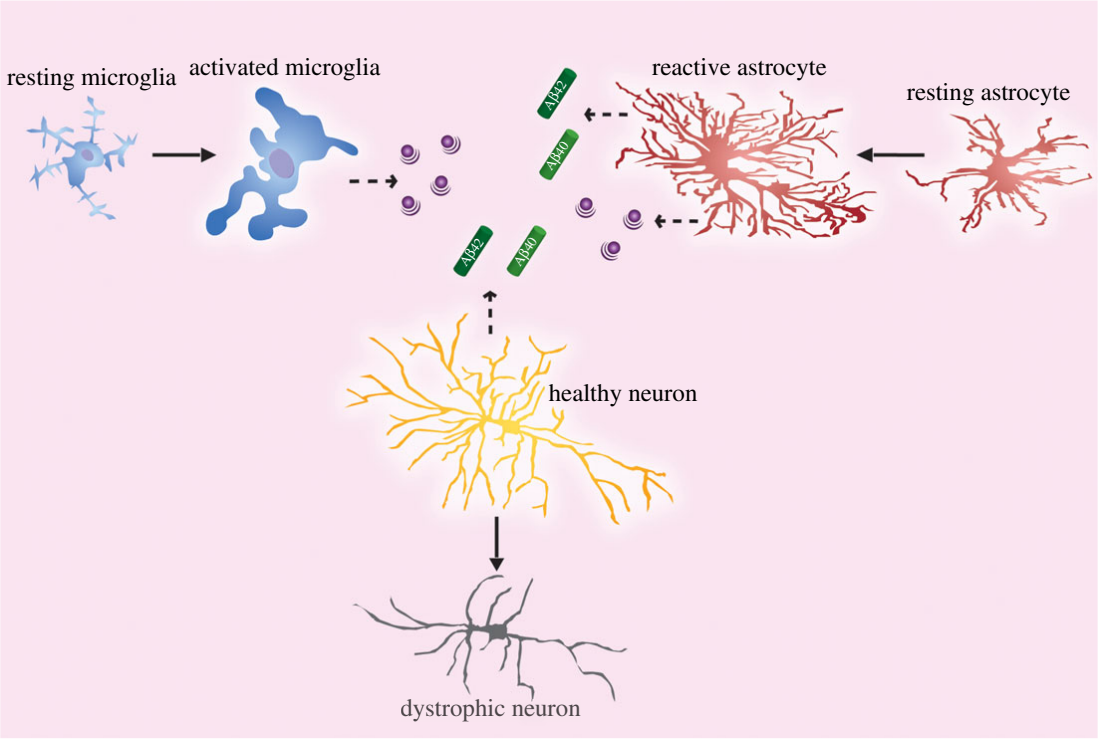
In AD, neurons secrete Aβ, which activates resting microglia and astrocytes. In turn, activated microglia and reactive astrocytes secrete proinflammatory mediators, which may induce neuronal death. Additionally, neuroinflammation also induces APP and BACE1 expression in astrocytes resulting in further Aβ production.

Taken together, this suggests a complicated relationship between neurons, microglia and astrocytes in the context of AD. Neurons contribute to total amyloid load, which consequently activates microglia and astrocytes. Microglia respond to Aβ by producing proinflammatory cytokines, which in turn activate astrocytes, inducing APP and BACE1 expression and further Aβ production. At the same time, astrocytes are also capable of clearing and degrading amyloid and secreting inflammatory mediators [216–218]. Downstream, inflammation may be a triggering event for the neuronal death seen in AD and contribute to cognitive decline (figure 4).

## 13. Conclusion

Several of the causal and risk factor genes for AD—amyloid precursor protein (APP), presenilin-1, presenilin-2, ApoE, clusterin (CLU), phosphatidylinositol-binding clathrin assembly protein (PICALM), triggering receptor expressed on myeloid cells 2 (TREM2)—are expressed not only by neurons but also, if not predominantly, by astrocytes [102], corroborating the idea that astrocytes are important players in AD pathogenesis. Neurons are often considered the lone source of Aβ in AD, yet there is plenty of evidence that astrocytes also contribute to Aβ load [111]. In particular, astrocytes activated by a multitude of cellular stressors upregulate the necessary machinery for Aβ production. This may be part of an innate immune response where Aβ functions as an AMP. Furthermore, astrocytes can be stimulated by Aβ from nearby neurons to make and secrete Aβ. In this cycle, Aβ-exposed astrocytes act as vectors to spread Aβ production in a self-sustaining way [219] that may drive AD pathology.

## Data accessibility

This article has no additional data.
